# Editorial: Artificial intelligence for data discovery and reuse in endocrinology and metabolism

**DOI:** 10.3389/fendo.2023.1180254

**Published:** 2023-05-05

**Authors:** Claudio Angione, Huajin Wang, Noël Burtt

**Affiliations:** ^1^ School of Computing, Engineering and Digital Technologies, Teesside University, North Yorkshire, United Kingdom; ^2^ Centre for Digital Innovation, Teesside University, North Yorkshire, United Kingdom; ^3^ National Horizons Centre, Teesside University, North Yorkshire, United Kingdom; ^4^ Open Science & Data Collaborations, Carnegie Mellon University, Pittsburgh, PA, United States; ^5^ Medical and Population Genetics Program and Metabolism Program, The Broad Institute of MIT and Harvard, Cambridge, MA, United States

**Keywords:** multi-modal, systems biology, multi-omic integration, mechanism & characterization, machine learning

## Introduction

As biomedical research has embraced the era of big data, massive amounts of complex multi-omic data are being generated. While there is huge potential in using the rich body of data to make new discoveries, many challenges exist in the dissemination, discovery, and reuse of these data. Artificial Intelligence (AI) and machine learning (ML) technologies have been paramount towards fully extracting value from rich and complex datasets to drive scientific discoveries and clinical decision-making. However, the adoption of AI and ML in endocrinology and metabolic diseases is lagging behind, compared to fields such as cancer genomics (1).

A major part of the challenge comes from the complexity and heterogeneity of data being produced by different omics platforms and research groups. In addition, there is a lack of data standards, data exchange platforms and data processing pipelines that are widely accepted by the community. Due to the complex and multi-faceted mechanisms underlying directly observable phenotypes, identifying multi-omic biomarkers that reflect the interplay between genetic regulation and metabolic response could provide novel insights into cellular functionality. Recent surveys have shown the role that metabolomic profiling plays in increasing the power of clinical variables, but have also highlighted its open challenges (2).

To fully leverage the power of AI to maximize the value of the rich data in endocrinology and metabolism, at least a few key areas need to be addressed. First, aggregated, harmonized, discoverable, and accessible datasets that are suitable for ML and AI applications are in urgent need, especially in the presence of multi-modal data. To this end, developing data-sharing infrastructure, standards, and data curation pipelines is the key to success. Second, it is becoming clear that incorporating mechanistic knowledge into ML and AI tools will facilitate a biologically-informed interpretation of the predictions. Third, it is crucial to develop easy-to-use tools and visualization methods that can be used by researchers and clinicians not trained as computer scientists to drive scientific discoveries and clinical decisions.

## Learning with multiple omic modalities

The articles presented in this Research Topic tackle the issue of learning with multiple omic modalities in a clinical context. Overall, they emphasise that the combination of multiple modalities is more effective than using only one modality in isolation, showing a significant increase in predictive performance. They also address the issue of small sample size, a common drawback of ML studies in omics, where obtaining matched samples across more than one modality remains a challenging task in terms of time and costs involved, with the associated challenges in using deep learning approaches (3, 4).

Feng et al. implement and compare eight ML models for the prediction of lateral lymph node metastasis in patients with papillary thyroid carcinoma, showing that random forest is highly effective and interpretable as a predictive method, but its performance is highly dependent on clinical variables. They also show that combining different modalities (clinical and sonographic in this case) improves the predictive performance.

Wu and Zhang apply various bioinformatics methods to identify differentially expressed genes, hub genes and signalling pathways that are potentially important for type 2 diabetes, using data from blood samples of subjects with type 2 diabetes vs healthy controls, downloaded from the GEO database. Further, a pharmacophore target analysis reveals potential drug target genes and pathways for celastrol, a natural phytochemical found to have anti-diabetic properties. The molecular interaction of celastrol and target genes is simulated by AlphaFold2.

Chen et al. study drug metabolism genes differentially expressed in human liver samples with or without NAFLD. Due to the small sample size, they reanalyse two publicly available GEO datasets, as well as previously-collected experimental data from the mouse, to enrich the main dataset and identify nine common differentially expressed genes.

Fu et al. compare five ML algorithms to predict the 52-week blood glucose level in 273 patients with type-2 diabetes, assessing the algorithms in terms of clinical and numerical performance measures. They conclude that XGBoost is the best choice to assist decision-making in the treatment of diabetic patients. They also discuss the challenges introduced by learning with a relatively small sample size.

Taken together, all studies show that focusing on interpreting the predictions generated by ML is a critical topic, especially for clinical applications (5–7). In this context, introducing mechanistic models within ML architectures is likely to represent a step change compared to existing data-driven approaches.

## Perspective: mechanism-aware and multi-modal machine learning

Data quality and data scarcity remain major challenges when dealing with the integration of multi-modal data through ML. Documentation, correct labelling and project metadata are as important as the data itself. Furthermore, dealing with missing data is a common issue among different data types or modalities, whether omic, imaging, or clinical data, which reduces the number of useable common samples. The limited number of matched samples in turn fuels the generalizability challenge, since a model with a limited number of samples tends to overfit the data. Transfer learning approaches can mitigate this issue.

It is also important to note that, in complex phenotypes where the interaction between events spanning multiple omic layers is likely to be the main cause of disease progression, traditional multi-omic computational methods based on ML are only able to uncover associations among genes, proteins or other omic components, without offering a mechanistic interpretation. As a result, these methods are not always able to provide the holistic understanding necessary to provide actionable biomarkers.

**Figure 1:**
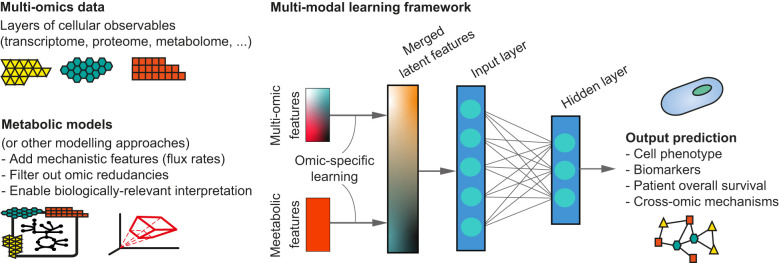
Example of a multi-modal learning framework combining omics data with mechanism-drive modelling approaches within a neural network architecture. In an intermediate integration strategy, multi-omics features and mechanistic model-derived features can be used for an independent phase of modality-specific training, before being fused in a single set of latent features. These can then be used within existing ML architectures (e.g. a fully connected neural network) to achieve the final prediction.

Therefore, new hybrid computational methodologies that are both data- and model-driven are needed for novel biomarker discovery, early diagnosis and better prediction of therapeutic targets (8, 9). For instance, multi-modal approaches to integrate multi-omic data with metabolic modelling (**Figure 1**) have shown promising results with higher accuracy and increased attention for the biological interpretation of ML-derived results (10–12). It seems therefore likely that combining different types of omics data with mechanism-driven models will further improve the ability of ML models to mechanistically characterize a disease.

Another potential direction is the direct incorporation of biological information within the learning process. This could be done by manually changing the structure of the ML architecture, or by adopting a combination of omics depending on the patient’s clinical characteristics, e.g. introducing an attention mechanism within the neural network (13). Biomarkers extracted from biologically-informed architectures are likely to have significantly higher potential for survival prognosis and therapeutic role compared to those generated *via* traditional model-agnostic interpretations.

## Author contributions

All authors listed have made a substantial, direct, and intellectual contribution to the work and approved it for publication.
